# Phenolic and fatty acid profile and antioxidant activity of *Fumaria agraria*

**DOI:** 10.1515/med-2026-1517

**Published:** 2026-09-08

**Authors:** Ferdaous Albouchi, Gianluigi Maria Lo Dico, Luay Rashan, Vittorio Calabrese, Manef Abderrabba

**Affiliations:** Laboratory of Materials Molecules and Applications, Preparatory Institute for Scientific and Technical Studies IPEST, University of Carthage, Carthage, Tunisia; Istituto Zooprofilattico Sperimentale della Sicilia “A. Mirri”, Palermo, Italy; Biodiversity Unit, Research Center, Dhofar University, Salalah, Oman; Department of Biomedical and Biotechnological Sciences, School of Medicine, University of Catania, Catania, Italy

**Keywords:** flavonoids, polyphenols, GC–FID, LC–MS/MS, radical scavenging

## Abstract

**Objectives:**

*Fumaria agraria* is still poorly characterized, although the interest in plant-derived bioactive compounds is growing. This study aimed to investigate its phytochemical composition and its antioxidant potential.

**Methods:**

Sequential solvent extraction yielded *n*-hexane, ethyl acetate and methanol fractions. Fatty acids were determined by gas chromatography with flame ionization detection (GC–FID) and phenolic compounds in the polar fractions by liquid chromatography–tandem mass spectrometry (LC–MS/MS). Antioxidant activity was assessed via DPPH, ABTS, and FRAP assays, alongside total phenolic (TPC) and flavonoid (TFC) content.

**Results:**

GC–FID analysis identified five fatty acids dominated by unsaturated forms (76.51 %), with linoleic acid as the principal individual component (40.29 %). LC–MS/MS analysis revealed 11 phenolic compounds, with luteolin being the most abundant phenolic compound, accounting for 67 % of the total identified polyphenols. The polar extracts exhibited the highest TPC, TFC, and antioxidant activity in all assays.

**Conclusions:**

*F. agraria* is a valuable source of unsaturated fatty acids and flavonoids, particularly luteolin, which likely contribute to the antioxidant activity of its polar extracts. These findings support its traditional medicinal use and highlight its potential as a source of natural antioxidant compounds, although further *in vivo* studies are required to confirm these results.

## Introduction

Traditional herbal medicine represents one of the earliest systems of healthcare. About half of all modern drugs are derived from medicinal plants, highlighting the critical role of botanical sources in pharmaceutical innovation and drug discovery. Tunisia, which is known for its remarkable floristic diversity, maintains a strong tradition of ethnomedicine based on plant remedies. The interest in phytotherapy has continuously increased mainly due to extensive pharmacological studies exploring the activity of bioactive plant compounds [1]. More than 6,000 plant species are currently used in traditional medicine, and about 2,500 of them have been reported to exhibit promising therapeutic potential against chronic diseases [2].

*Fumaria agraria* is an annual herbaceous species of the genus *Fumaria*, which includes about 60 species distributed mainly in the Mediterranean region [3]. Plants from this genus are well recognized for their medicinal properties and are commonly used in folk medicine. Indeed, their extracts are reported to possess hepatoprotective, analgesic, anti-inflammatory, antiviral, antioxidant, antimicrobial and even antitumor properties. Traditional infusions prepared from the aerial parts are often used as diuretics and antihypertensives agents, and also for the relief of ailments such as arthritis, gallstones and skin disorders [4], 5].

Phytochemical analysis has shown that *Fumaria* species are rich in alkaloids, flavonoids, and phenolic acids. These compounds contribute to their biological activities and explain their long use for digestive health, liver detoxification and dermatological disorders [4], [5], [6], [7]. It is important to note that the accurate identification and the standardization of these compounds are essential to develop safe and effective plant-derived therapies, which is only possible through detailed pharmacognostic and analytical studies [8]. The recent progress of GC–MS and LC–MS has greatly improved the profiling of plant metabolites. Indeed, GC–MS presents high reproducibility for low-molecular-weight compounds, whereas LC–MS is more adapted to semi-polar or non-volatile compounds such as flavonoids, terpenoids and phenolic acids [9], [10], [11]. Among these techniques, LC–MS/MS remains indispensable in plant metabolomics because it allows the discrimination of structurally similar molecules [12], 13].

Several *Fumaria* species present documented biological effects [4], *F. agraria* remains poorly characterized. Moreover, the available data on the genus are inconsistent and vary according to the plant part, the geographic origin and the extraction method [3], 4], 7]. This absence of consensus justifies a systematic and method-standardized characterization of the individual species, instead of an extrapolation across the whole genus. We therefore hypothesized that *F. agraria* contains significant levels of phenolic compounds and unsaturated fatty acids that are responsible for its antioxidant activity. To verify this hypothesis, we combined LC–MS/MS and GC–FID analyses, which provide one of the first complete phytochemical and antioxidant characterizations of this species.

## Materials and methods

### Chemicals and reagents

All reagents used were analytical or HPLC–grade. Ultrapure water (18.2 MΩ cm) was obtained from a Milli-Q^®^ Integral system with a Q pod (Millipore, Bedford, MA, USA). Acetic acid, acetonitrile and 2-propanol were purchased from VWR International Srl. (Milan, Italy); hydrochloric acid and sodium hydroxide were obtained from Carlo Erba (Milan, Italy). HPLC–grade methanol was purchased from Merck (Darmstadt, Germany). Standard solutions of vanillic acid, trans-ferulic acid, myricetin, naringenin, pinocembrin, luteolin, and hesperidin were purchased from Extrasynthese (Genay Cedex, France). Chlorogenic acid was obtained from HWI Analytik GmbH (Rülzheim, Germany). Gallic acid, ellagic acid, caffeic acid, apigenin, quercetin, kaempferol, rutin, catechin and epicatechin were obtained from Sigma-Aldrich Srl. (Milan, Italy).

### Plant material and extract preparation

The aerial parts of *F. agraria* were collected during the vegetative stage in northwest Tunisia (Bousalem, Jendouba governorate). Botanical identification was performed by Professor Mohamed Boussaid, Head of the Laboratory of Plant Biotechnology at the National Institute of Applied Sciences and Technology (INSAT, Tunis, Tunisia), and a voucher specimen was deposited in the Herbarium of the Department of Botany under accession number 1471. Plant collection did not require specific permits, as *F. agraria* is neither a protected nor an endangered species in Tunisia. The harvested aerial parts were dried in a dark and ventilated environment at room temperature (20 ± 2 °C) to preserve their bioactive compounds. The dried plant material was ground into a fine powder using an electric grinder.

Ten grams of dried and pulverized aerial parts of *F. agraria* were subjected to sequential extraction using solvents of increasing polarity (*n*-hexane, ethyl acetate, and methanol) to recover non-polar, semi-polar and polar metabolites, respectively. Water and ethanol were deliberately excluded to minimize the co-extraction of sugars, proteins, and other highly polar constituents that could interfere with subsequent LC–MS/MS and GC–FID analyses [14]. The plant material was first defatted by maceration in *n*-hexane (100 mL, 48 h, under orbital agitation, three times); the combined extracts were filtered through Whatman No. 1 filter paper and evaporated under reduced pressure at 40 °C. The defatted residue was then sequentially extracted with ethyl acetate and methanol (100 mL each, 10 min sonication followed by 24 h of maceration, three times), filtered, combined, and concentrated at 40 °C. Extraction yield (EY%) was calculated as:

(1) $$EY\left(\%\right)=\frac{M_{1}-M_{0}}{m}\times 100$$

where *M*_1_ is the weight (g) of the dry residue combined with the round-bottomed flask after vacuum evaporation; *M*_0_ is the weight (g) of the empty round-bottomed flask; and *m* is the weight (g) of the dry plant material used. Extracts were stored in amber vials at 4 °C for up to one month.

### Phytochemical analysis

#### Phytochemical screening

The total phenolic and flavonoid contents were determined as previously described [15]. For TPC, 500 µL of a diluted extract was mixed with 5 mL of Folin–Ciocalteu reagent (1:10), and 4 mL of a 7 % sodium carbonate solution, incubated in the dark (15 min) at room temperature, and absorbance was measured at 765 nm against a reagent blank.

For TFC, 500 µL of diluted extract was mixed with 1.5 mL of methanol, 0.1 mL of 10 % AlCl_3_ solution, 0.1 mL of 1 M potassium acetate, and 2.8 mL of distilled water, incubated at room temperature for 30 min, and absorbance was recorded at 415 nm against a reagent blank. Gallic acid and quercetin were used as reference standards for TPC and TFC, respectively. TPC was expressed as milligrams of gallic acid equivalents per g of dry extract (mg GAE/g DE), while TFC was expressed as milligrams of quercetin equivalents per g of dry extract (mg QE/g DE).

#### Fatty acid composition

The composition of *F. agraria* fatty acid methyl esters was determined according to the method previously described by [16]. This method involves the convertion of the lipid extract to fatty acid methyl esters. Briefly, 1 mL of *n*-hexane was added to 40 mg of the *n*-hexane extract, followed by the addition of 200 μL of sodium methoxide (2 M). The mixture was heated at 50 °C for 20 s, followed by the addition of 200 μL of HCl (2 M). One μL of the top layer was collected and analyzed using analytical gas chromatography (GC) (Agilent 6890N, California, USA) equipped with a flame ionization detector (FID) and a polar capillary column (HP-Innowax 0.25 mm internal diameter, 30 m length and 0.25 µm film thickness). The detector temperature was set at 275 °C, and the column temperature was maintained at 150 °C for 1 min, then increased at a rate of 15 °C/min to 200 °C, and at a rate of 2 °C/min to 250 °C and held for 4 min. The fatty acid methyl esters peaks were identified by comparing their retention times with individual standards. The relative percentage of each fatty acid was calculated based on the maximum area of the fatty acid species relative to the total peak area of all the fatty acids in the lipophilic sample [16].

#### Polyphenol composition

The phenolic compounds were analyzed as previously described [17]. Polyphenols were identified and quantified using a Transcend II System with Multiplexing and TurboFlow Technology (Dionex – Thermo Fisher Scientific) coupled to a Q–Exactive Plus Hybrid Quadrupole-Orbitrap Mass Spectrometer (Thermo Fisher Scientific) equipped with a Heated Electrospray Ionization (HESI) source operating in negative ionization mode.

Sample purification was achieved using a Cyclone-P column (50 mm × 0.5 mm, 60 μm particle size, 60 Å pore size, Thermo Fisher Scientific), while chromatographic separation was performed using a Hypersil Gold column (2.1 mm × 100 mm, 1.7 μm particle size). The mobile phase consisted of the following eluents: A, 30 mmol/L ammonium acetate (pH 5); B, methanol; C, water (0.5 % formic acid); and D, acetonitrile/acetone/2-propanol (4:3:3). Eluents A and B were used to optimize chromatographic resolution, while eluents B, C, and D were employed for TurboFlow online purification. The extracts and standards were dissolved in eluent A at a 1:10 dilution (v/v). The injection volume was 5 μL, and elution was carried out at a flow rate of 0.2 mL/min with the following gradient program: 0–2 min, 95 % A and 5 % B; 4 min, 60 % A and 40 % B; 6 min, 0 % A and 100 % B; 9 min, 0 % A and 100 % B; 11.5 min, 95 % A and 5 % B; 14 min, 95 % A and 5 % B; 18 min, 95 % A and 5 % B.

The total running time was 18 min. In negative polarity mode, the eluted compounds were detected by MS with the following ion source settings: sheath gas flow rate of 35 arbitrary units (AU); auxiliary gas flow rate of 10 AU; spray voltage of 3.50 kV; capillary temperature at 300 °C; tube lens voltage of 55 V; heater temperature of 305 °C; scan mode, full scan; scan range (*m*/*z*), 100–700; microscans, 1; negative-mode resolution, 35,000; AGC target of 1 × 10^6^; and maximum IT of 100 ms. The chromatographic parameters were as follows: column temperature, 30 °C; sample temperature, 6 °C. The autosampler holder temperature was maintained at 7 °C. Data acquisition and processing were performed using Thermo Scientific Xcalibur software (version 4.0) with Qual Browser and Quan Browser modules.

Phenolic compound identification was based on retention times, accurate mass measurements, and MS/MS fragmentation patterns. Where available, identification was further confirmed using authentic reference standards and comparison with published literature data. Quantification was performed using individual calibration curves for vanillic, caffeic, ferulic, and ellagic acids, hesperidin, myricetin, luteolin, quercetin, rutin, apigenin, and kaempferol. Results were expressed as μg/kg dry weight (DW).

Compounds with signals below the limit of quantification were reported as <LOQ, whereas compounds without detectable signals were reported as ND (not detected).

### Antioxidant activity

DPPH and ABTS radical scavenging activities, as well as reducing power (FRAP), were evaluated using the method described by Albouchi et al. [15].

#### DPPH radical scavenging assay

The free radical scavenging activity of the extracts against DPPH (2,2-diphenyl-1-picrylhydrazyl) was evaluated, with slight modifications. Briefly, 1 mL of a methanolic extract solution (0.01–1 mg/mL) was mixed with 2 mL of freshly prepared DPPH methanol solution (0.1 mM). The mixture was gently shaken and incubated in the dark at room temperature for 15 min. The absorbance was then measured at 515 nm using a UV–vis spectrophotometer. The radical scavenging activity was calculated as the percentage decrease in absorbance relative to the DPPH control. Butylated hydroxytoluene (BHT) and Trolox were used as reference antioxidants. The scavenging activity was calculated according to the equation:

(2) $$\text{SA}\left(\%\right)=\left(\frac{A_{0}-A_{s}}{A_{0}}\right)\times 100$$

where SA is the scavenging activity (%), *A*_0_ is the absorbance of the control (DPPH solution without sample), and *A*_*s*_ is the absorbance of the sample.

#### ABTS assay

The 2,2′-azino-bis(3-ethylbenzothiazoline-6-sulfonic acid) ABTS^·+^ radical cation was produced by mixing equal volumes of 7 mM ABTS solution and 2.45 mM potassium persulfate, and allowing the mixture to stand in the dark at room temperature for 12–16 h. The ABTS^·+^ working solution was diluted with methanol to obtain an absorbance of 0.70 ± 0.02 at 734 nm. Then, 1 mL of extract solution (0.01–1 mg/mL) was added to 2 mL of the ABTS^·+^ solution, mixed thoroughly, and left to react for 6 min in the dark. Absorbance was measured at 734 nm using a UV–vis spectrophotometer. Trolox was used as a standard, and results were expressed as percentage inhibition of the ABTS radical, calculated as:

(3) $$Inhibition\left(\%\right)=\frac{A_{0}-A_{s}}{A_{0}}\times 100$$

where *A*_0_ and *A*_*s*_ are the absorbances of control and sample, respectively.

#### Ferric reducing antioxidant power (FRAP) assay

The FRAP reagent was freshly prepared by combining 300 mM acetate buffer (pH 3.6), 10 mM TPTZ (2,4,6-tripyridyl-s-triazine) dissolved in 40 mM HCl, and 20 mM FeCl_3_ · 6H_2_O in a 10:1:1 (v/v/v) ratio. A standard calibration curve was constructed using FeSO_4_ · 7H_2_O, and the antioxidant capacity was expressed as micrograms of FeSO_4_ equivalents per gram (μg FeSO_4_/g). For the *n*-hexane extract, the absorbance recorded in the FRAP assay did not differ significantly from the reagent blank, indicating a reducing capacity below the detection limit of the method; this extract was therefore reported as ND (not detected) rather than as a numerical value.

### Statistical analysis

Extraction was performed in triplicate for each solvent. Analytical determinations were performed in three repetitions for all the phytochemical and antioxidant assays; the results are expressed as mean ± SEM. Statistical analyses were performed using one-way analysis of variance (ANOVA) for multiple group comparisons and Student’s t-test for paired data. The Pearson correlation, principal component analysis (PCA) and hierarchical cluster analysis (HCA) were based on only three combined extracts (n=3; 1 degree of freedom). Therefore, this analysis is considered a descriptive tool rather than an inferential one: with this number of extracts, only a perfect coefficient (|*r*| = 1.00) reaches p<0.05, and the two PCA axes necessarily explain 100 % of the variance (n − 1=2 axes). All analyses were carried out in R (version 4.0.5, R Core Team, Vienna, Austria), with statistical significance fixed at (p<0.05).

## Results

### Determination of phenolic compound content

The sequential scheme with three solvents (*n*-hexane, ethyl acetate, methanol) separates the plant constituents according to the “like dissolves like” principle. Indeed, *n*-hexane recovers the lipophilic compounds (hydrocarbons, waxes, terpenoids, fatty acids), ethyl acetate recovers the semi-polar constituents (flavonoid aglycones, phenolic acids), and methanol recovers the hydrophilic compounds (glycosides and other polar phenolics) [14], [18], [19], [20], [21], [22].

**Figure 1:**
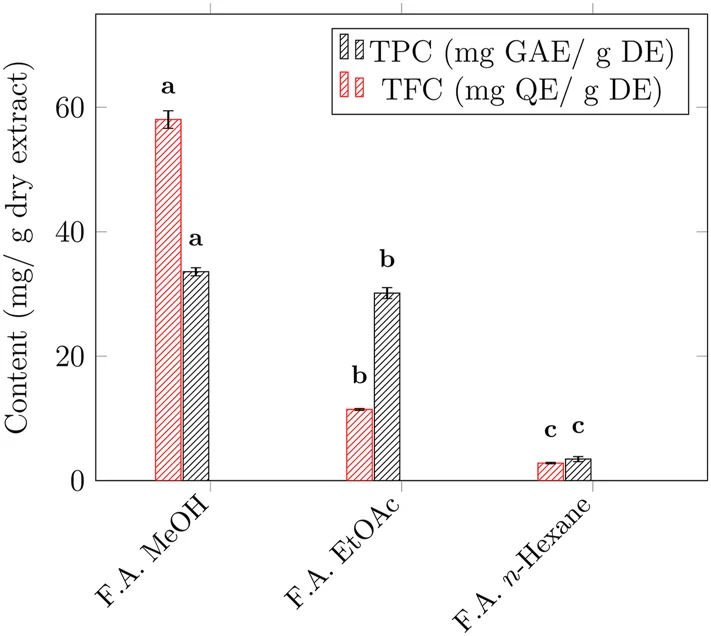
Total phenolic content (TPC) and total flavonoid content (TFC) of *Fumaria agraria* organic extracts. Data are presented as mean ± SEM of technical triplicate (n=3) of combined extract per solvent. Bars sharing the same lowercase letter within the same parameter are not significantly different (p<0.05, one-way ANOVA followed by Tukey’s post hoc test).

The TPC and the TFC from *F. agraria* aerial part extracts are shown in Table 1 and Figure 1. The results show that TPC and TFC depend on the extraction solvent. The methanol extract exhibited the highest TPC and TFC (33.58 ± 0.65 mg GAE/g DE and 58.04 ± 1.41 mg QE/g DE, respectively), followed by the EtOAc extract (30.15 ± 0.87 mg GAE/g DE and 11.44 ± 0.15 mg QE/g DE, respectively). The lowest values were obtained with the *n*-hexane extract.

**Table 1:** Total phenolics and flavonoids contents in *Fumaria agraria* organic extracts.

*F. agraria* organic extracts	Extraction yields, %	Total phenolic, mg GAE/g DE	Total flavonoid, mg QE/g DE
F.A. MeOH	2.9 ± 0.24^a^	33.58 ± 0.65^a^	58.04 ± 1.41^a^
F.A. EtOAc	1.78 ± 0.54^a^	30.15 ± 0.87^b^	11.44 ± 0.15^b^
F.A. *n*-Hexane	5.1 ± 0.4^c^	3.46 ± 0.40^c^	2.82 ± 0.11^c^

F.A. MeOH, methanolic extract of *F. agraria*; F.A. EtOAc, ethyl acetate extract of *F. agraria*; F.A. *n*-Hexane, *n*-hexane extract of *F. agraria*. Total phenolic content (TPC) was expressed as milligrams of gallic acid equivalents per gram of dry extract (mg GAE/g DE), whereas total flavonoid content (TFC) was expressed as milligrams of quercetin equivalents per gram of dry extract (mg QE/g DE). Data are presented as mean ± SEM of three replicates (n=3). Different superscript letters within the same column indicate statistically significant differences between extracts at p<0.05, as determined by one-way ANOVA followed by Tukey’s post hoc test.

This dependence on the solvent is in agreement with the reports on related species (*Fumaria capreolata, Fumaria officinalis, Fumaria jankae, Fumaria rostellata, Fumaria indica, Fumaria vaillantii*), for which the phenolic content varies according to the species, the plant part, the developmental stage and the extraction conditions [3], 5], 23], 24].

The TFC of the methanol extract (58.04 mg QE/g DE) was higher than its TPC (33.58 mg GAE/g DE), which may seem paradoxical due to flavonoids are a subclass of polyphenols. However, this difference is not real and is explained by methodological factors. First, the AlCl_3_ method can overestimate the flavonoid content because it also reacts with other compounds sharing similar structural features, such as certain phenolic acids and tannins [25]. Second, the reference standards have different molar masses (quercetin 302.24 vs. gallic acid 170.12 g/mol), which increases the numerical value of the TFC. Similar observations were reported for other plant extracts [23].

### Characterization of phenolic compounds by LC–MS/MS

**Figure 2:**
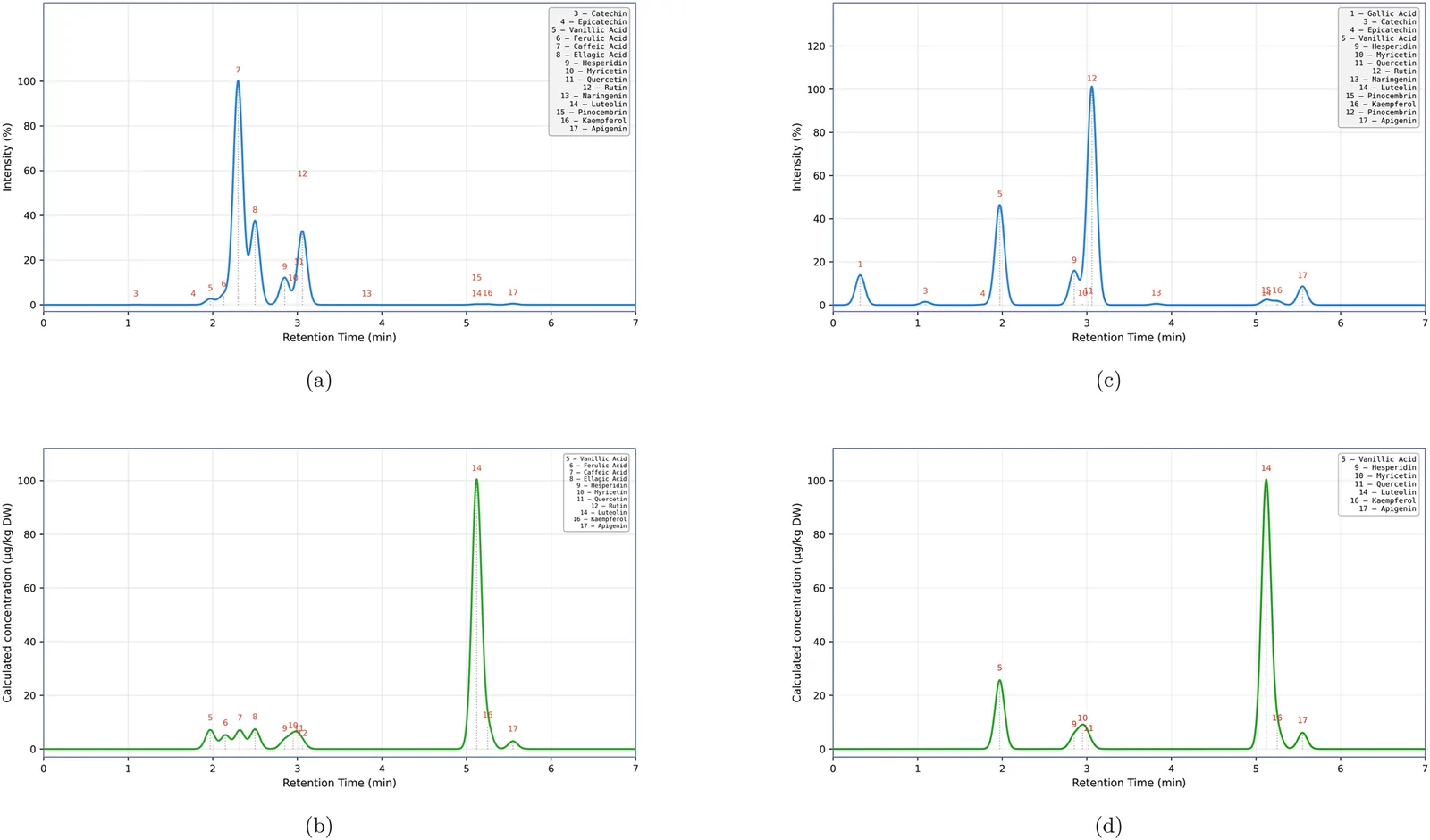
LC–MS/MS chromatographic profiles of *Fumaria agraria* methanolic (A) and ethyl acetate (B) extracts. (a, c) Raw signal chromatograms showing instrumental intensity (a.u.) vs. retention time, with each numbered peak corresponding to a phenolic compound identified by its characteristic *m*/*z*. (b, d) Reconstructed concentration chromatograms (μg/kg dry weight, DW) derived from external calibration curves. Only analytes above the limit of detection (LOD) and within the calibration range are shown; compounds flagged as not detected (ND) or below the limit of quantification (<LOQ) are excluded. Peak numbering refers to Table 2.

**Figure 3:**
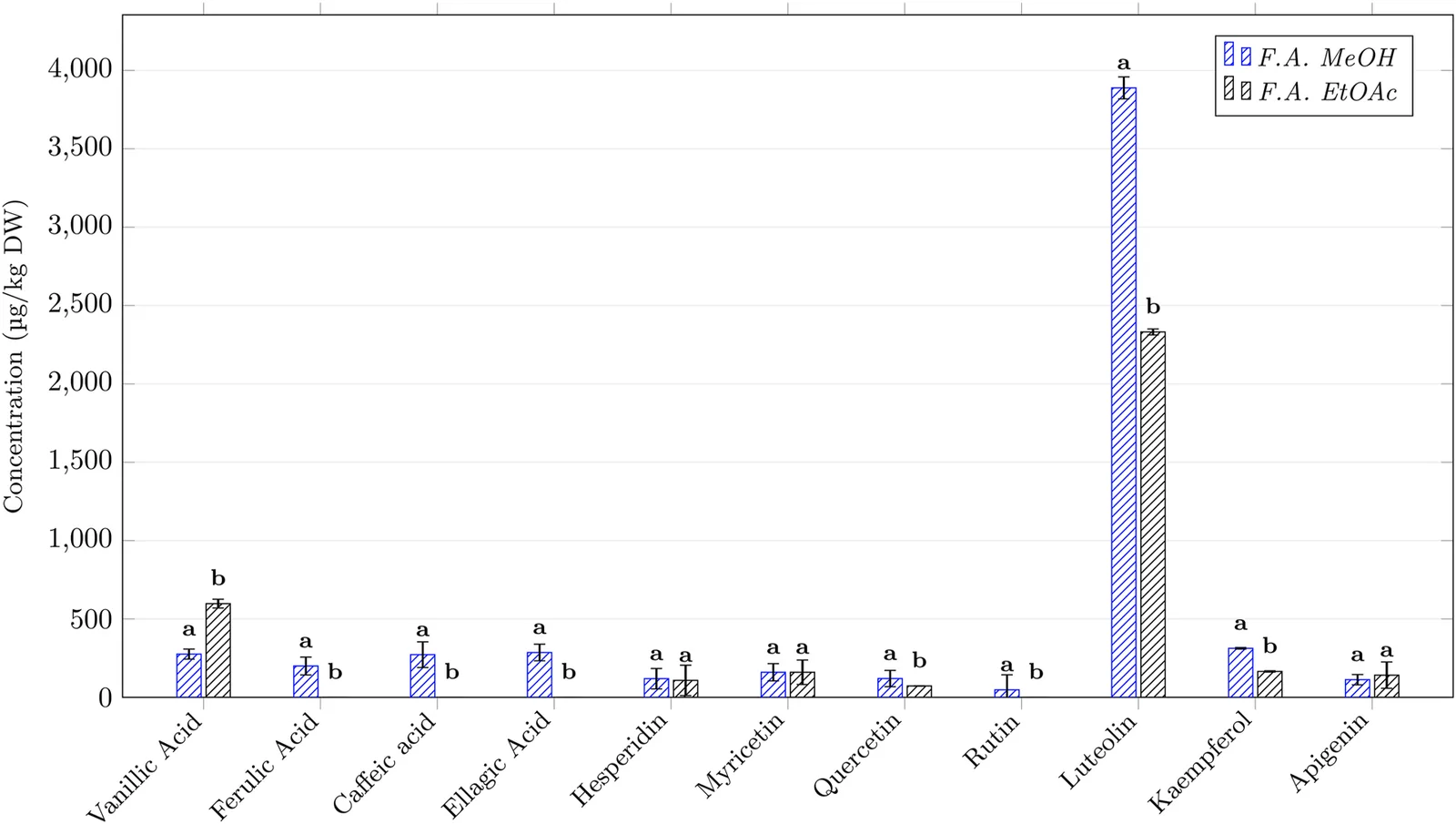
Phenolic compound profiles of methanolic and ethyl acetate extracts of *F. agraria*. Compound concentrations were quantified by LC–MS/MS and expressed as μg/kg dry weight (DW), presented as mean ± SEM of technical triplicate (n=3) of combined extract per solvent. Different letters above each pair of bars indicate statistically significant differences between extracts, as determined by one-way analysis of variance (ANOVA) followed by Tukey’s post hoc test at p<0.05.

The LC–MS/MS analysis identified and quantified 11 phenolic compounds, using the retention times, the [M−H]^−^ (*m/z*) and the MS/MS fragmentation patterns (Figure 2, Table 2); six other compounds were ND or<LOQ. The two polar extracts were dominated by flavonoids (82 % and 83 % of the identified compounds in MeOH and EtOAc, respectively), and luteolin was the main compound (67 % and 65 %). The retention times ranged between 1.97 and 5.55 min in negative mode. The MeOH extract contained four phenolic acids (vanillic, caffeic, ferulic, ellagic) and seven flavonoids (rutin, quercetin, hesperidin, myricetin, luteolin, kaempferol, apigenin), whereas the EtOAc extract contained only one phenolic acid (vanillic) and six flavonoids (quercetin, hesperidin, myricetin, luteolin, kaempferol, and apigenin). This significant difference is explained by the different polarities of the two solvents (Figure 3). Luteolin was present at a higher concentration in MeOH than in EtOAc (3,888.78 vs. 2,331.40 μg/kg DW), and on the contrary vanillic acid was higher in EtOAc (597.76 vs. 275.71 μg/kg DW). In comparison with the previous report on *F. agraria*, which described a profile rich in protocatechuic acid and caffeic acid [7], our data show substantial qualitative and quantitative differences.

**Table 2:** Phenolic composition of methanolic and ethyl acetate extracts of *Fumaria agraria* analyzed by LC–MS/MS.

Peak	RT, min	Fragments (*m*/*z*)	C.A.S. No.	Formula	Compound	Family	Concentration, μg/kg DW	
							F.A. MeOH	F.A. EtOAc
1	0.32	125.00	149-91-7	C_7_H_6_O_5_	Gallic acid		ND	<LOQ
2	0.68	191.20	327-97-9	C_16_H_18_O_9_	Chlorogenic acid	Phenolic acid	ND	ND
3	1.09	203.20	154-23-4	C_15_H_14_O_6_	Catechin		<LOQ	<LOQ
4	1.77	203.20	490-46-0	C_15_H_14_O_6_	Epicatechin	Flavanols	<LOQ	<LOQ
5	1.97	108.88	121-34-6	C_8_H_8_O_4_	Vanillic acid		275.71 ± 20.32^a^	597.76 ± 27.28^b^
6	2.13	143.00	1135-24-6	C_10_H_10_O_4_	Ferulic acid		199.76 ± 11.57^a^	ND
7	2.30	135.02	331-39-5	C_9_H_8_O_4_	Caffeic acid	Phenolic acid	272.07 ± 20.82^a^	ND
8	2.50	131.98	476-66-4	C_14_H_6_O_8_	Ellagic acid		286.00 ± 11.53^a^	ND
9	2.85	301.00	520-26-3	C_28_H_34_O_1_5	Hesperidin		118.72 ± 15.65^a^	108.00 ± 4.97^a^
10	2.95	151.00	529-44-2	C_15_H_10_O_8_	Myricetin		159.84 ± 8.55^c^	159.84 ± 11.78^c^
11	3.02	151.00	117-39-5	C_15_H_10_O_7_	Quercetin		119.65 ± 6.52^a^	72.47 ± 4.1^b^
12	3.06	300.30	207671-50-9	C_27_H_30_O_16_	Rutin		47.72 ± 1.96^a^	<LOQ
13	3.82	119.00	480-41-1	C_15_H_12_O_5_	Naringenin	Flavonoid	<LOQ	<LOQ
14	5.12	133.00	491-70-3	C_15_H_10_O_6_	Luteolin		3,888.78 ± 147.7^a^	2,331.40 ± 96.19^b^
15	5.12	151.00	480-39-7	C_15_H_12_O_4_	Pinocembrin		<LOQ	<LOQ
16	5.25	108.00	520-18-3	C_15_H_10_O_6_	Kaempferol		312.48 ± 26.05^a^	164.98 ± 13.04^b^
17	5.55	117.00	520-36-5	C_15_H_10_O_5_	Apigenin		112.5 ± 20.33^a^	141.45 ± 10.84^a^

RT, retention time; F.A. MeOH, methanolic extract of *F. agraria*; F.A. EtOAc, ethyl acetate extract of *F. agraria*; ND, not detected; <LOQ, below the limit of quantification. Compound concentrations were quantified by LC–MS/MS and expressed as μg/kg dry weight (DW) as mean ± SEM values of technical triplicate (n=3) of combined extract per solvent. Different superscript letters within the same row indicate statistically significant differences between extracts at p<0.05, according to one-way ANOVA followed by Tukey’s post hoc test.

### GC–FID analysis of fatty acids

The *n*-hexane extract was rich in unsaturated fatty acids (Table 3). Notably, oleic (27.17 %), linoleic (40.29 %) and *α*-linolenic (9.05 %) acids, together represented 76.51 % of the total fatty acids, while the saturated acids were represented by palmitic (16.09 %) and stearic (4.01 %) acids. These five fatty acids represented 96.61 % of the total, and the remaining 3.39 % corresponded to trace or unidentified peaks. A previous qualitative study had identified four fatty acids, two fatty acid esters and two fatty alcohols in *F. agraria* and related species [26]; the present work completes these data with a detailed quantitative GC–FID profile. In the related species *Fumaria parviflora*, the main fatty acids reported were linoleic, myristic, lignoceric, palmitic, arachidonic, decanoic, lauric and oleic acids [27].

**Table 3:** Fatty acid composition of *Fumaria agraria n*-hexane extract.

Peak number	Retention time, min	Fatty acid	Carbon length	^f^Composition, %
1	4.96	Palmitic	C16:0	16.09 ± 0.03^a^
2	5.99	Stearic	C18:0	4.01 ± 0.01^b^
3	7.69	Oleic	C18:1	27.17 ± 0.72^c^
4	13.05	Linoleic	C18:2	40.29 ± 1.15^d^
5	17.90	*α*-Linolenic	C18:3	9.05 ± 0.8^e^
		^g^SAFA	–	20.10
		^h^MUFA	–	27.17
		^i^PUFA	–	49.34
		^j^P/S	–	2.45

^f^The relative percentage of each fatty acid was calculated from its peak area relative to the total peak area of identified fatty acids in the *n*-hexane extract; ^g^MUFA: monounsaturated fatty acids; ^h^PUFA: polyunsaturated fatty acids; ^i^SAFA: saturated fatty acids; ^j^P/S: ratio of polyunsaturated to saturated fatty acids. Data are expressed as mean ± SEM of technical triplicate (n=3) of combined extract per solvent. Different superscript letters within the same column indicate statistically significant differences at p<0.05, as determined by one-way ANOVA followed by Tukey’s post hoc test.

### Antioxidant activity

**Figure 4:**
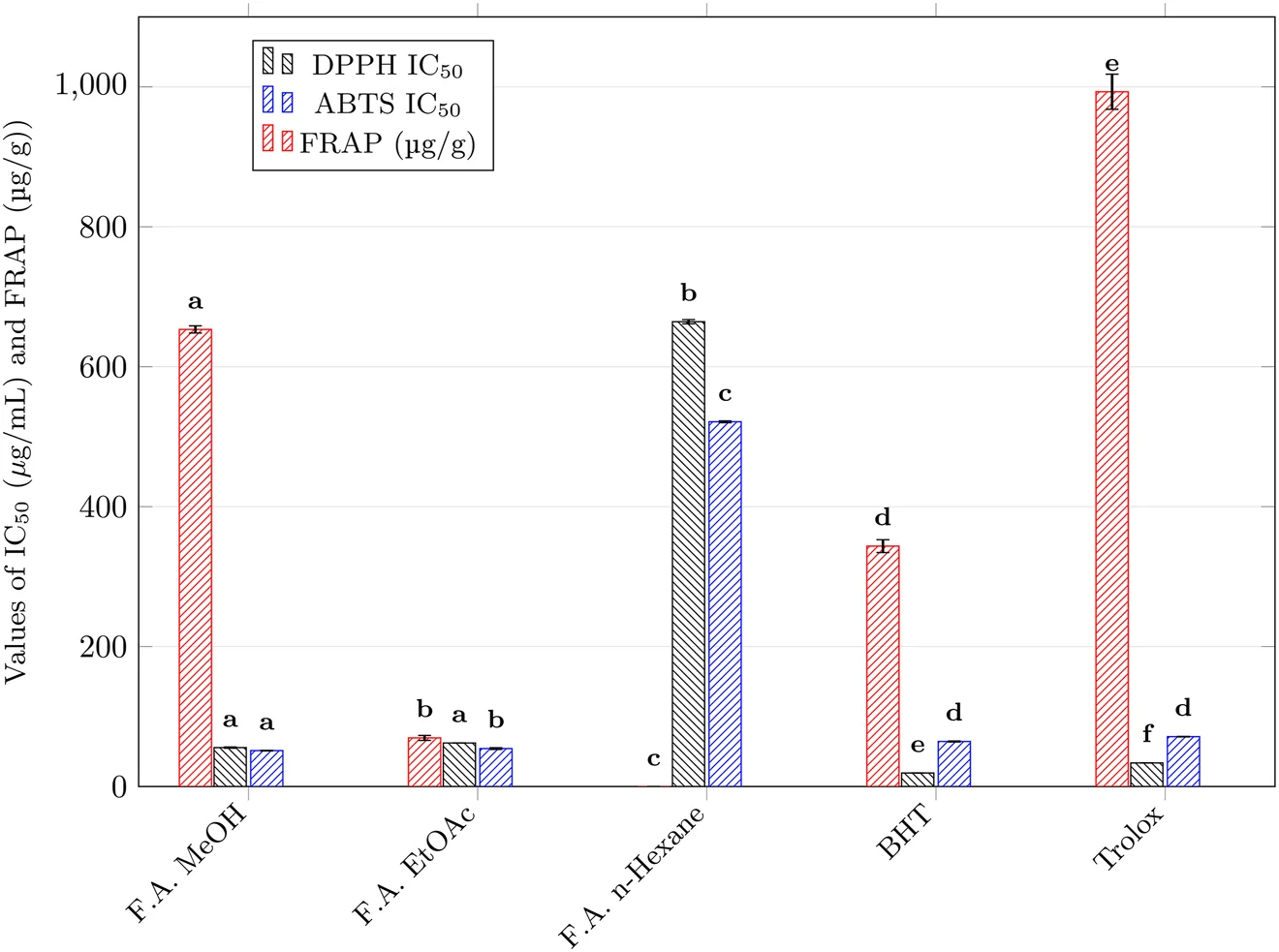
Antioxidant activities of *Fumaria agraria* organic extracts (methanolic, ethyl acetate, and *n*-hexane) and reference antioxidants (BHT and Trolox), evaluated using DPPH and ABTS radical scavenging assays as well as ferric reducing antioxidant power (FRAP). DPPH and ABTS results are expressed as IC_50_ values (μg/mL), whereas FRAP values are expressed as μg FeSO_4_/g. Data are presented as mean ± SEM of technical triplicate (n=3) of combined extract per solvent. Different superscript letters above bars indicate statistically significant differences between samples at p<0.05, according to one-way ANOVA followed by Tukey’s post hoc test.

**Figure 5:**
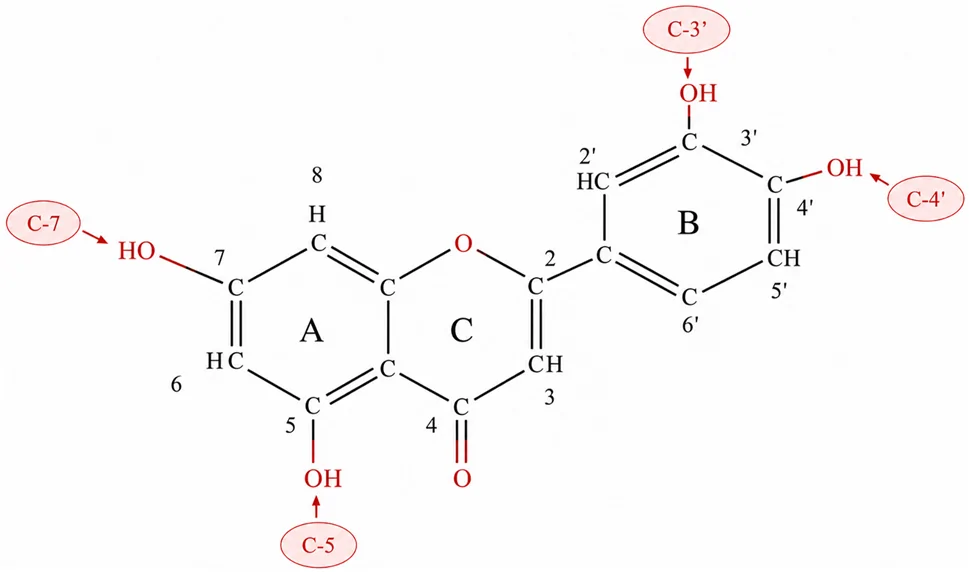
Chemical structure of luteolin (3′,4′,5,7-tetrahydroxyflavone). The molecule consists of three fused rings (A, B, and C) with a carbonyl group (C=O) at position C-4. Four hydroxyl groups (–OH) are highlighted in red at positions C-5 and C-7 on ring A, and C-3′ and C-4′ on ring B.

As shown in Table 4 and Figure 4, all the extracts showed a concentration-dependent activity. The methanolic extract neutralized the DPPH and ABTS radicals (IC_50_ 55.61 ± 0.91 μg/mL and 51.36 ± 0.43 μg/mL, respectively), followed by the ethyl acetate extract (62.24 ± 0.11 and 54.25 ± 1.21 μg/mL, respectively). The *n*-hexane extract was clearly weaker with IC_50_ values of 664.32 ± 3.02 and 521.32 ± 1.29 μg/mL, respectively. In the ABTS assay, the methanolic extract presented lower IC_50_ values than both reference antioxidants, BHT and Trolox, which may be due to its high luteolin content (Figure 5).

**Table 4:** DPPH, ABTS assay and ferric reducing antioxidant power FRAP of *Fumaria agraria* extracts.

*F. agraria* organic extracts	DPPH IC_50_, μg/mL	ABTS IC_50_, μg/mL	FRAP (μg FeSO_4_/g)
F.A. MeOH	55.61 ± 0.91^a^	51.36 ± 0.43^a^	653.32 ± 5.14^a^
F.A. EtOAc	62.24 ± 0.11^b^	54.25 ± 1.21^a^	69.50 ± 3.52^b^
F.A. *n*-Hexane	664.32 ± 3.02^c^	521.32 ± 1.29^b^	ND
BHT	19.33 ± 0.18^d^	64.37 ± 0.74^c^	343.61 ± 9.18^c^
Trolox	33.7 ± 0.07^e^	71.28 ± 0.35^d^	993.04 ± 25.10^d^

F.A. MeOH, methanolic extract of *F. agraria*; F.A. EtOAc, ethyl acetate extract of *F. agraria*; F.A. *n*-Hexane, *n*-hexane extract of *F. agraria*; ND, not detected (below the detection limit of the assay). FRAP values are expressed as μg FeSO_4_/g, whereas DPPH and ABTS antioxidant activities are expressed as IC_50_ values (μg/mL). All results are presented as mean ± SEM of technical triplicate (n=3) of combined extract per solvent. Different superscript letters within the same column indicate statistically significant differences between samples at p<0.05, according to one-way ANOVA followed by Tukey’s post hoc test.

The FRAP value was the highest for the methanol extract (653.32 ± 5.14 μg FeSO_4_/g), significantly higher than that of the ethyl acetate extract (69.5 ± 3.52 μg FeSO_4_/g). The *n*-hexane extract did not show any detectable reducing power. These results indicate that polar extracts act as effective scavengers and as efficient reducing agents. This activity is due to their phenolic composition and is explained by hydrogen- and/or electron-donating mechanisms [15]. Our results are in agreement with those of Soušek et al., who limited their evaluation of the *F. agraria* polar extracts to the tBHP-induced lipoperoxidation and the DPPH scavenging [7].

### Correlation

**Figure 6:**
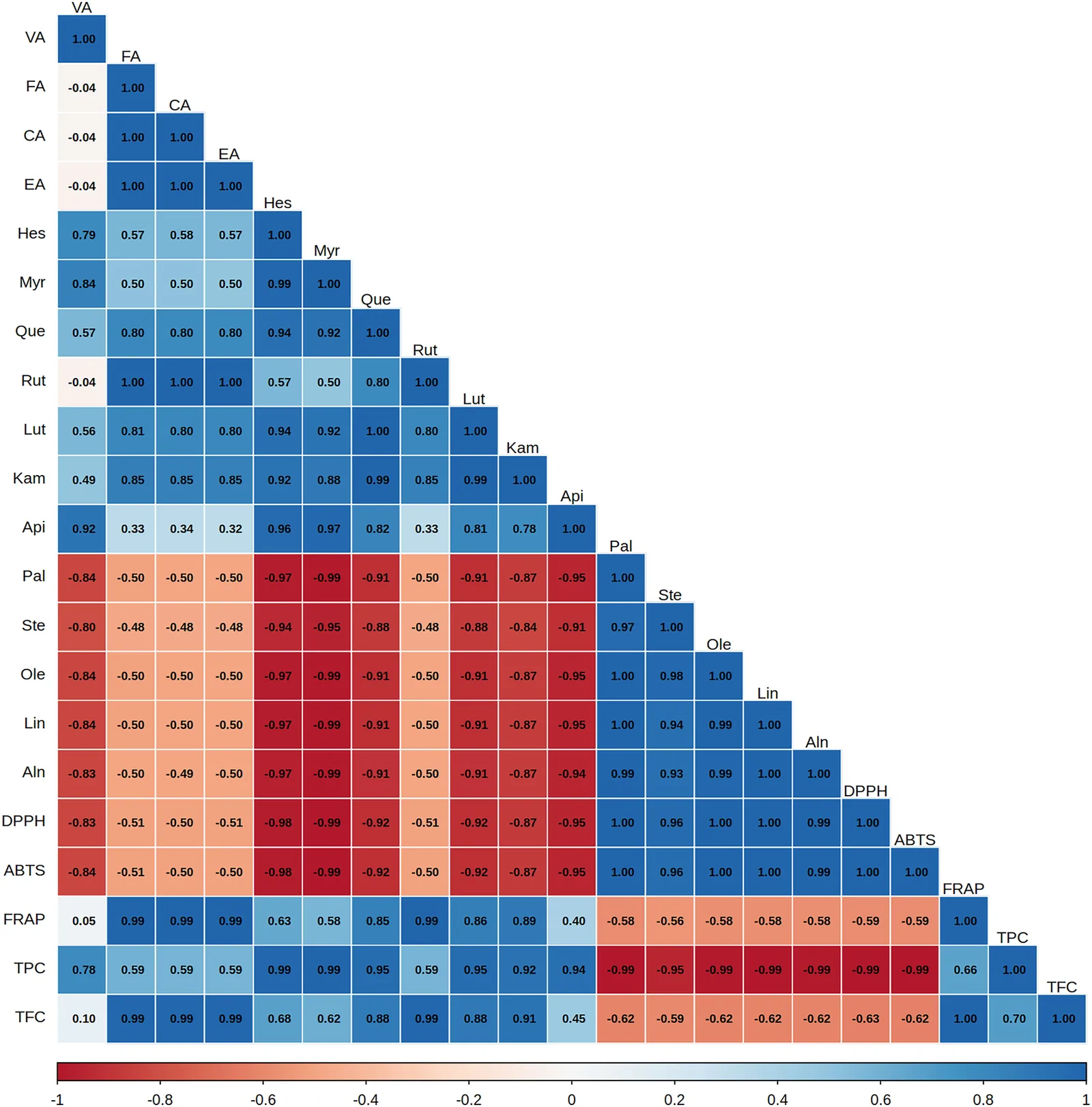
Pearson correlation matrix between TPC, TFC, phenolic compounds, fatty acids and antioxidant activities of *Fumaria agraria* organic extracts. VA: vanillic acid; FA: ferulic acid; CA: caffeic acid; EA: ellagic acid; Hes: hesperidin; Myr: myricetin; Que: quercetin; Rut: rutin; Lut: luteolin; Kam: kaempferol; Api: apigenin; Pal: palmitic acid; Ste: stearic acid; Ole: oleic acid; Lin: linoleic acid; Aln: *α*-linolenic acid; DPPH and ABTS expressed as IC_50_ (μg/mL); FRAP expressed as μg FeSO_4_/g.

Pearson correlation analysis was performed to evaluate the relationships between TPC, TFC, the identified phenolic and fatty acid compounds, and the antioxidant activities (DPPH, ABTS, FRAP) of *F. agraria* organic extracts. As displayed in Figure 6, TPC exhibited strong negative correlations with DPPH and ABTS IC50 (*r* = −0.99 for both), as well as a moderate positive correlation with FRAP (*r* = +0.66). Similarly, TFC showed negative correlations with DPPH IC50 (*r* = −0.63) and ABTS IC50 (*r* = −0.62), and a near-perfect positive correlation with FRAP (*r* = +1.00), the latter being the only coefficient approaching the statistical significance threshold given the sample size.

At the level of individual compounds, strong negative correlations with DPPH and ABTS IC_50_ values were observed for several compounds, notably myricetin (*r* = −0.99), hesperidin (*r* = −0.98), apigenin (*r* = −0.95), luteolin (*r* = −0.92), and quercetin (*r* = −0.92), followed by kaempferol (*r* = −0.87). Regarding ferric reducing power (FRAP), near-perfect positive correlations were observed with ferulic acid (*r*=0.99), caffeic acid (*r*=0.99), ellagic acid (*r*=0.99), and rutin (*r*=0.99), highlighting the key role of phenolic acids and glycosides in the reducing capacity of the extracts. In contrast, the fatty acids identified in the *n*-hexane extract exhibited strong positive correlations with DPPH IC50 and ABTS IC50 values (palmitic acid: *r*=1.00; oleic acid: *r*=1.00; linoleic acid: *r*=1.00; stearic acid: *r*=0.96), and a negative correlation with the FRAP (*r* = −0.58), reflecting the markedly lower capacity of this extract relative to the polar fractions. In general, these trends show that the phenolic compounds, and particularly the flavonoids, are the major contributors to the antioxidant potential of *F. agraria* extracts.

### Principal component analysis and cluster analysis

**Figure 7:**
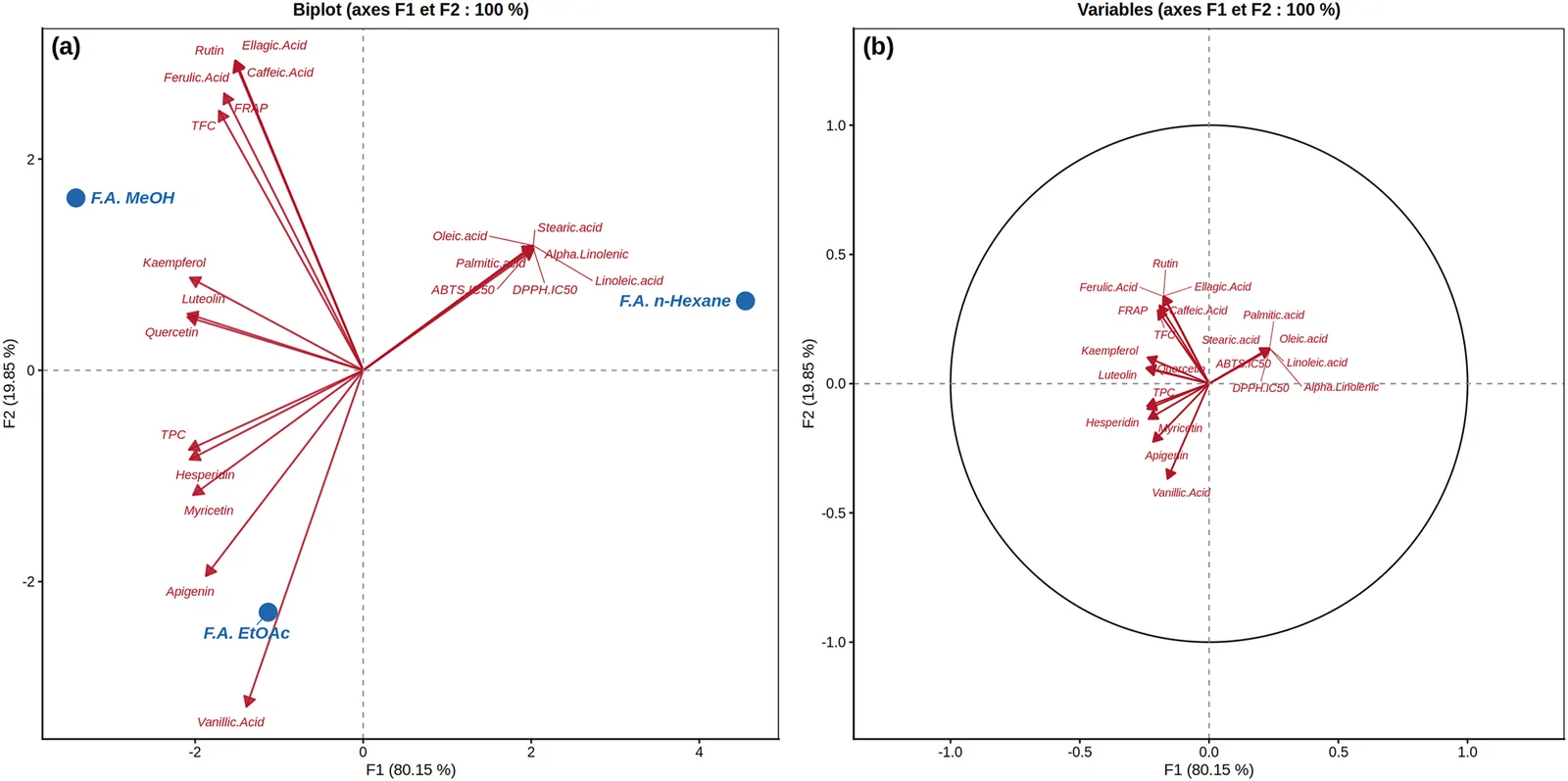
Principal component analysis (PCA) of phytochemical constituents, fatty acid composition, and antioxidant activities of *Fumaria agraria* organic extracts. (a) PCA biplot showing the distribution of methanolic (F.A. MeOH), ethyl acetate (F.A. EtOAc), and *n*-hexane (F.A. *n*-Hexane) extracts according to the measured variables. The projection highlights the relationships between extracts and bioactive compounds, including phenolic acids, flavonoids, fatty acids, total phenolic content (TPC), total flavonoid content (TFC), and antioxidant activities (DPPH, ABTS, and FRAP). (b) Correlation circle illustrating the contribution and correlation of variables with the first two principal components (F1 and F2), which together explained 100 % of the total variance (F1=80.15 %, F2=19.85 %). Variables located close to the circle are strongly correlated with the principal components, whereas variables grouped in the same direction indicate positive correlations.

**Figure 8:**
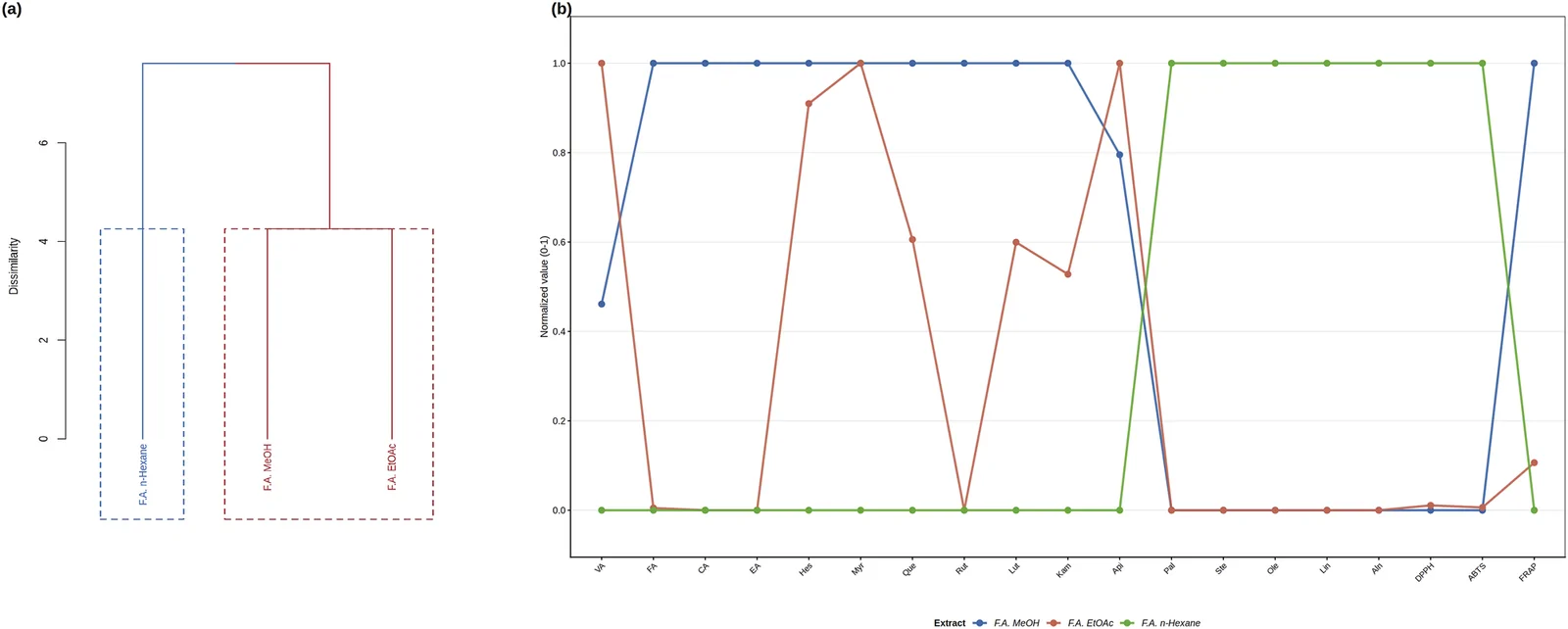
Hierarchical clustering and phenotypic characterization of phytochemical and antioxidant diversity in *Fumaria agraria* organic extracts. (a) Hierarchical cluster analysis (HCA) dendrogram showing the similarity relationships among *F. agraria* organic extracts based on their phytochemical composition and antioxidant activities. (b) Parallel coordinate plot representing the normalized distribution patterns of phenolic compounds, fatty acids, and antioxidant activities (DPPH, ABTS, and FRAP) in methanolic (F.A. MeOH), ethyl acetate (F.A. EtOAc), and *n*-hexane (F.A. *n*-Hexane) extracts of *F. agraria*. Data were normalized on a scale ranging from 0 to 1 to facilitate comparative visualization among extracts.

Principal component analysis (PCA) and hierarchical cluster analysis (HCA) were performed on the phenolic compounds, fatty acids, and antioxidant activities of *F. agraria* extracts to provide an integrative visualization of the relationships between variables and extracts (Figures 7 and 8, Tables 5–7). The PCA was applied to 21 variables across the three extracts. The first two components explained all the variance (F1=80.15 %, F2=19.85 %; Table 5). This complete explanation of the variance is a direct consequence of the small number of extracts (n=3), so the biplot is considered a descriptive visualization only. The axis F1 separated the two polar extracts (negatively correlated with luteolin, quercetin, TPC, hesperidin, kaempferol, myricetin, TFC, FRAP and apigenin; Table 6) from the *n*-hexane extract (positively correlated with the five fatty acids and with the DPPH and ABTS IC_50_; Table 6). It is important to note that the IC_50_ is inversely proportional to the antioxidant activity. Therefore, the DPPH/ABTS vectors point towards the *n*-hexane extract only because this extract dominates the variance of these variables (IC_50_ about 10 times higher); this reflects its low activity and not a strength. The axis F2 separated the two polar extracts (Table 6): the methanol extract was characterized by caffeic, ellagic and ferulic acids, rutin and FRAP, and the ethyl acetate extract by vanillic acid (597.76 ± 27.28 vs. 275.71 μg/kg DW in F.A. MeOH) and apigenin (Table 6). The correlation circle (Figure 7b) revealed three groups of variables: the phenolic acids with the FRAP (methanol extract), vanillic acid with apigenin (ethyl acetate extract), and the fatty acids with the high IC_50_ (*n*-hexane extract). The HCA (Ward’s method, Euclidean distance) revealed two groups (Figure 8a). The first group was the *n*-hexane extract, clearly separated because of its profile dominated by fatty acids and poor in phenolics. The second group gathered the methanol and ethyl acetate extracts, because of their similar profiles rich in flavonoids and their comparable radical-scavenging activities (DPPH IC_50_ 55.61 and 62.24 μg/mL). However, the two polar extracts also presented differences, notably for vanillic acid (higher in EtOAc) and for the absence of caffeic acid, ferulic acid, ellagic acid and rutin in the EtOAc fraction. The parallel coordinate plot (Figure 8b) confirms this structure: the methanol extract presented the highest normalized values for the phenolics and the FRAP, the ethyl acetate extract presented a more selective profile with a maximum for vanillic acid, and the *n*-hexane extract presented a maximum only for the fatty acids. Overall, solvent polarity was the main factor governing composition and antioxidant activity of the tested extracts.

**Table 5:** Variance explained by the principal axes of the PCA.

Axis	Eigenvalue (*λ*)	Variance, %	Cumulative variance, %	Interpretation
F1	16.83	80.15	80.15	Main discrimination axis
F2	4.17	19.85	100.00	Secondary differentiation axis

**Table 6:** Correlations and contributions of variables on the first two principal component axes (F1 and F2) of the PCA performed on phenolic compounds, fatty acids, and antioxidant activities of *Fumaria agraria* organic extracts.

Variable	*r*, F1	*r*, F2	Contribution F1, %	Contribution F2, %
DPPH IC_50_	+0.963	+0.270	5.51	1.74
ABTS IC_50_	+0.962	+0.274	5.50	1.80
Palmitic acid	+0.960	+0.279	5.48	1.86
Stearic acid	+0.960	+0.279	5.48	1.86
Oleic acid	+0.960	+0.279	5.48	1.86
Linoleic acid	+0.960	+0.279	5.48	1.86
*α*-Linolenic acid	+0.960	+0.279	5.48	1.86
TPC	−0.984	−0.177	5.76	0.75
TFC	−0.814	+0.580	3.94	8.07
Vanillic acid	−0.659	−0.752	2.58	13.55
Ferulic acid	−0.722	+0.692	3.09	11.49
Caffeic acid	−0.722	+0.692	3.09	11.49
Ellagic acid	−0.722	+0.692	3.09	11.49
Rutin	−0.722	+0.692	3.09	11.49
Hesperidin	−0.980	−0.200	5.71	0.95
Myricetin	−0.960	−0.279	5.48	1.86
Quercetin	−0.993	+0.119	5.86	0.34
Luteolin	−0.992	+0.126	5.85	0.38
Kaempferol	−0.978	+0.207	5.69	1.03
Apigenin	−0.888	−0.460	4.69	5.06
FRAP	−0.785	+0.619	3.66	9.19

TPC, total phenolic content; TFC, total flavonoid content; DPPH, 2,2-diphenyl-1-picrylhydrazyl; ABTS, 2,2′-azino-bis(3-ethylbenzothiazoline-6-sulfonic acid); FRAP, ferric reducing antioxidant power; IC_50_, half-maximal inhibitory concentration. Variables in the upper block are positively correlated with F1 and characterize the *n*-hexane extract; variables in the lower block are negatively correlated with F1 and characterize the polar extracts (F.A. MeOH and F.A. EtOAc). Given the limited number of extracts (n=3), these results are presented as a descriptive/integrative tool rather than an inferential one.

**Table 7:** Factorial coordinates of the three *Fumaria agraria* organic extracts.

Extract	F1	F2	Biochemical profile
F.A. MeOH	−3.42	+1.63	Rich in phenolic acids (caffeic, ellagic, ferulic, rutin), high TFC, best FRAP activity (653.32 ± 5.14 μg/g)
F.A. EtOAc	−1.13	−2.29	Dominated by vanillic acid and apigenin; moderate TPC; intermediate antioxidant activity
F.A. *n*-Hexane	+4.55	+0.66	Exclusively fatty acids (linoleic, oleic, palmitic); very high DPPH/ABTS IC_50_=low antioxidant activity

## Discussion

In the present study, we found that *F. agraria* is rich in phenolic compounds and unsaturated fatty acids, which together contributed significantly to its antioxidant activity as assessed by DPPH, ABTS, and FRAP assays. The flavonoids accounted for approximately 82 % and 83 % of the total identified phenolic compounds of the MeOH and EtOAc extracts, respectively. This proportion is consistent with previous phytochemical surveys that reported an enrichment in flavonoids in the polar extracts of *Fumaria* species [3], 7], 23], 24], and with the known efficiency of the polar solvents for the recovery of the phenolic compounds [14], 28]. The polyphenols and fatty acids exhibit a large range of biological activities, namely antimicrobial, antioxidant, anti-inflammatory, immunomodulatory, and anticancer effects, and are able to modulate signaling pathways involved in oxidative stress and inflammation, including NF-*κ*B, MAPK, PI3K/AKT, and prostaglandin cascades [29], [30], [31], [32], [33], [34], [35], [36]. Indeed, several studies have demonstrated that an inability to manage reactive oxygen species, such as superoxide radicals $\left({\text{O}}_{2}^{•-}\right)$ and hydrogen peroxide (H_2_O_2_), leads to oxidative damage to cellular components such as proteins, membrane lipids, and DNA, thereby promoting inflammation and contributing to the development of a multitude of diseases. From a mechanistic standpoint, the antioxidant properties of polyphenols are related to their molecular structure and their capacity to donate electrons or hydrogen atoms to neutralize radical species, to act via classical chain-breaking mechanisms, and to chelate transition metals [37].

Contrary to most previous chemotaxonomic studies of *Fumaria* species, which emphasized the isoquinoline alkaloid fraction as the genus’s chemotaxonomic marker [6], our LC–MS/MS analyses identified luteolin as the dominant phenolic compound of the *F. agraria* polar extracts (67 % of total identified compounds). This finding is differs from reports in which quercetin derivatives or rutin were the major flavonoid constituents [3], 23], which suggests variability at the species level, probably linked to geographic origin, extraction conditions, or plant developmental stage.

Structurally, luteolin (3′,4′,5,7-tetrahydroxyflavone) belongs to the flavone subclass and possesses four hydroxyl groups (-OH) at positions 5, 7, 3′, and 4′ of its flavone skeleton, as shown in Figure 5. These hydroxyl groups confer strong reducing and antioxidant properties. Indeed, luteolin inhibits the formation of ROS and activates antioxidant defenses (glutathione, catalase, superoxide dismutase) [38], which is in agreement with its reported anti-inflammatory, antimicrobial, chemopreventive, cardioprotective, anti-diabetic, neuroprotective and anti-allergic activities [39], [40], [41], [42], [43]. The polar extracts also contained vanillic, ferulic, caffeic and ellagic acids, and the flavonoids quercetin, hesperidin, myricetin, kaempferol, apigenin and rutin. It is worth noting that quercetin and kaempferol suppress NF-*κ*B activation and modulate MAPK signaling, while hesperidin and apigenin down-regulate adhesion molecules [29], [44], [45], [46], [47]. The combined action of these polyphenols, with luteolin as the major contributor, probably increases the antioxidant efficacy of the polar extracts. In the ABTS assay, the polar extracts were more active than Trolox and BHT, which indicates a stronger radical-scavenging activity than these synthetic antioxidants. However, this superiority must be interpreted with caution. The IC_50_ values were expressed on a mass basis and not on a molar basis. Therefore, the mixture of phenolics of the extract, in which each compound acts at a low individual molar concentration, probably produces this activity by a combined or synergistic action, and not by the potency of a single compound. Moreover, the ABTS radical is less sterically hindered than the DPPH radical, so it reacts more easily with polyhydroxylated flavonoids. This explains why the superiority was observed only in the ABTS assay, while BHT and Trolox remained more active in the DPPH assay, in agreement with the assay-dependent differences reported for luteolin-rich extracts [48]. It is also important to note that the benefit of the phenolics *in vivo* depends on their bioavailability. In general, these compounds are poorly absorbed and reach high concentrations in the intestinal lumen, where they can act as anti-inflammatory, anti-apoptotic, anti-carcinogenic and anti-proliferative agents [49], 50].

The lipophilic (*n*-hexane) extract presented a distinct profile dominated by unsaturated fatty acids (76.51 %), and particularly by linoleic acid (40.29 %). These fractions rich in fatty acids were less studied in the genus *Fumaria* than the alkaloid and flavonoid fractions [26], 27]. Their presence indicates that *F. agraria* is a species that combines hydrophilic (flavonoid-based) and lipophilic (fatty-acid-based) antioxidant mechanisms, representing a dual antioxidant strategy that is not always reported together in a single *Fumaria* species. The unsaturated fatty acids, and especially linoleic and oleic acids, act mainly by modulating MAPK signaling and by suppressing oxidative stress [51], [52], [53], [54].

The correlation trends are in agreement with the literature [55], [56], [57], [58]. The strong negative correlations of the TPC with the DPPH and ABTS IC_50_ (*r* = −0.99) show that the phenolics are the main radical scavengers. These observations are similar to the negative correlations between the polyphenols, the flavonoids and the antioxidant activity reported in green tea [55], to the strong relationship between the phenolics and the scavenging activity in spent grains (*r*=0.975) [56], and to the marked correlations across the FRAP, DPPH and chelating assays in *Hovenia acerba* *Lindl* [57].

At the level of the individual compounds, the flavonoids identified in *F. agraria* extracts, notably myricetin, hesperidin, apigenin, luteolin and quercetin, showed strong negative correlations with the DPPH and ABTS IC_50_ values (*r* ranging from −0.92 to −0.99). This observation may be attributed, at least in part, to the chemical structures of these molecules, since the structural characteristics of flavonoids, and particularly the number and the position of their hydroxyl groups, are widely recognized as important determinants of antioxidant activity [59], [60], [61], [62]. Overall, these correlations are consistent with the antioxidant properties of flavonoids previously reported in the literature [48], [59], [60], [61], [62], [63]. The near-perfect correlations of the FRAP with the TFC and with ferulic acid, caffeic acid, ellagic acid and rutin (*r*≥0.99) show that the phenolic acids and the glycosides are the main drivers of the reduction, in agreement with the observations reported in *Cistus salviifolius* [64] and in green tea [55]. The weak or inconsistent correlations obtained for some phenolic acids (for example, vanillic acid) are explained by the assay-specific mechanisms and by the composition of each extract, which depends on the solvent, as noted in other studies [55], 65].

The PCA and HCA results corroborate the correlation analysis, confirming that solvent polarity is the principal factor governing both the phytochemical composition and the antioxidant capacity of the *F. agraria* extracts, in a manner comparable to the solvent- or genotype-driven clustering reported for other plant matrices [56], 63]. This convergence between the multivariate and correlation analyses further supports the well-established relationship between phenolic content and antioxidant activity [28], 29].

This study presents some limitations. First, the antioxidant activity was evaluated only *in vitro*, so its physiological relevance must still be established. Second, the stability of the extracted compounds and the possible synergistic or antagonistic interactions were not assessed. Third, the reported triplicates reflect the technical variability. Finally, the study was focused on a single plant part (aerial parts) and a single growth stage (vegetative stage), which may not represent the whole chemical diversity of the species. Because the number of extracts is small (n=3), the multivariate results are descriptive and must be confirmed with larger datasets.

## Conclusions

To the best of our knowledge, this study is among the first to combine LC–MS/MS and GC–FID profiling with a multi-assay antioxidant evaluation of *F. agraria* extracts. In conclusion, the phenolic compounds and the fatty acids of its extracts act through complementary mechanisms, including radical scavenging and ferric ion reduction, which together support the antioxidant capacity of the polar fractions, with luteolin as the main contributor. Considering the growing interest in natural alternatives to synthetic antioxidants such as BHT, *F. agraria* represents a promising source of bioactive molecules that deserve further investigation, in particular *in vivo*.
